# “Let me tell you what I think about online psychological help.” A thematic analysis of voluntary opinions collected at the onset of the COVID-19 pandemic

**DOI:** 10.3389/fpsyg.2023.1141856

**Published:** 2023-07-17

**Authors:** Arkadiusz Wesołowski, Julianna Skawińska, Emilia Soroko

**Affiliations:** Faculty of Psychology and Cognitive Science, Adam Mickiewicz University in Poznań, Poznań, Poland

**Keywords:** online psychological help, online psychotherapy, attitudes toward online psychological help, thematic analysis, qualitative method, e-mental health, COVID-19, pandemic

## Abstract

**Introduction:**

The COVID-19 pandemic shifted many aspects of life from face-to-face to an online form, including psychological help. Many people had to face the choice of adjourning contact with a psychologist or shifting it to the Internet. This study aimed to develop an understanding of attitudes and opinions toward relatively new phenomenon in Poland – online psychological help.

**Method:**

Seventy two (*N*=72) statements about relationship between COVID-19 pandemic and online psychological help from (potential) patients were included in this research. The statements were collected from a community sample via open-ended question for volunteers added to an online survey conducted regarding an existing project. The statements were exclusively written responses to the following question: *If you want to provide us with something about the relationships between the COVID-19 pandemic and online help/psychotherapy, please let us know below.* By reason of exploratory character of our study and general phenomenological philosophical approach and constructionist approach, a thematic analysis method was used to analyze the data.

**Results:**

The analysis led us to identify three general themes with sub-themes that refer to meaningful aspects of online psychological help: 1. Online psychological help situates in the shadow of face-to-face help, 1.1. It frustrates the needs, especially the need for psychological contact, 1.2. It contributes to negative emotions, 1.3. It is sometimes better than the face-to-face help; 2. Online psychological help is a solution during the COVID-19 pandemic, 2.1. It provides a sense of continuity during lockdown, 2.2. It is a means to adapt to exceptional circumstances, 3. The concerns about the credibility and effectiveness of online psychological help.

**Discussion:**

The results show (potential) patients’ attitudes (including emotions, thoughts, and concerns) toward online psychological help. The perspective presented here could be beneficial to professionals. A better understanding of client/patient attitudes will allow for more accurate customization of the online help and sensitize psychologists to the emotions that may occur about online psychological help. It could also be beneficial for patients to understand how other people would feel about online psychological help and develop ones’ own self-awareness of the attitudes toward online psychological help.

## Introduction

Technological advances have contributed to the movement of most areas of life, such as work, study, and health, into the online realm. Psychotherapy and all other forms of psychological help are subjected to technology, resulting in a plethora of modern healthcare information technologies such as mobile apps, online exercise programs, and therapeutic interventions (Hayes and Hofmann, 2020). Some forms of online psychological help are used as pure self-help, while others require regular contact with a psychologist via the Internet (Clarke et al., 2009). The majority of the literature indicates that online psychotherapy is as effective as its face-to-face counterpart (Wagner et al., 2014). Meta-analyses show small-to-medium effect sizes when Internet interventions are delivered as stand-alone self-help interventions, and medium-to-large effect sizes when delivered as therapist-guided interventions, both compared with usual care (Schröder et al., 2016). However, the results of meta-analyses are not always so conclusive, especially when studying improvements from specific disorders (White et al., 2022), suggesting potentially limited real-world effectiveness and the need to specify the conclusions that are most favorable to successful online psychological help. In addition to this, online psychological help has undoubted advantages, such as being able to help people in rural areas who may find it challenging to access psychotherapy physically (Simpson and Reid, 2014). Online interventions are also easily disseminated and are low-cost (Clarke et al., 2009).

The natural pace of moving psychotherapy from face-to-face reality to virtual space has been accelerated by the COVID-19 pandemic. The pandemic and resulting lockdowns reduced social contact and complications at work, thus creating problems not only in terms of psychological difficulties but also in the availability of face-to-face psychological help (Vindegaard and Benros, 2020). The global situation has also forced therapists to move from face-to-face to online therapy for the most acute period of the pandemic. Considering the Polish psychological help field at the onset of the COVID-19, therapists did not receive unified guidelines for organizing online support. Consequently, at the onset of the pandemic, they had to find a path to provide support in times of restrictions. Some psychologists had to decide on their own whether to switch to remote contact or suspend therapy (Zielona-Jenek et al., 2021). Some associations, e.g., the Polish Society for Psychodynamic Psychotherapy (Polskie Towarzystwo Psychoterapii Psychodynamicznej, 2020), released the statement at the beginning of the pandemic in which they did not recommend conducting therapy in an online form. However, PTPPd pointed out some specific areas of therapeutic work that can be conducted online (especially support in crisis and maintaining a therapeutic relationship). Moreover, government online psychological support programs targeting specific groups began to appear, e.g., the “Comfort Zone” of the Parliament of Students of the Republic of Poland (“Strefa Komfortu” Parlamentu Studentów Rzeczpospolitej Polskiej) as well as online help provided by companies (e.g., WeTalk Chat). Thus, at the pandemics’ beginning, online psychological support in Poland was organized multidirectionally without clear legal rules or government recommendations. The abrupt transition time, often combined with a lack of preparation or experience in the online contact form, was also associated with many difficulties (Guinart et al., 2021; Lewis et al., 2021; Zielona-Jenek et al., 2021). Literature suggests that managing the transition to online psychological help depends on the psychotherapy approach; specifically, therapists identifying with the cognitive-behavioral approach presented a more positive attitudes than psychodynamics psychotherapists. One possible explanation for this difference is the emphasis on different mechanisms of change in each therapy. Psychodynamic psychologists focus more on, e.g., in-session processes, non-verbal responses, and working through silence, which may be limited, distorted, or even impossible from the perspective of conducting therapy in an online form. (Békés and Aafjes-van Doorn, 2020; Hayes and Hofmann, 2020). Moreover, some authors focused on non-professional and professional helpers point out that non-professionals rate telemedicine more critically (Schulze et al., 2019). Professionals are likely affected by the exposure effect. The literature provides the pros and cons of online help experiences stated by practitioners’. Feijt et al. (2020) mainly reported technical (e.g., unstable connection) or communication (e.g., lack of non-verbal signs) issues and unpleased needs (e.g., technical/procedural support or sufficient resources) as limitations of online help. Moreover, psychologists’ negative attitudes were also related to the quality of the therapy, such as perceiving lower competence, lower confidence, and lower authenticity of the therapeutic relationship (Békés and Aafjes-van Doorn, 2020). However, convenience in making appointments, providing additional information about a client, improved contact (e.g., some clients are less inhibited) (Feijt et al., 2020) and previous experiences of online therapy, and beliefs of a positive patient experience (Békés and Aafjes-van Doorn, 2020) are considered as advantages of online psychological help.

The literature suggests that people would rather choose face-to-face than online therapy. However, they do not exclude the possibility of using remote appointments. Patients prefer face-to-face therapy rather than online therapy (e.g., Wong et al., 2018), even when all advantages related to online help are considered (Rochlen et al., 2004; Klein and Cook, 2010). March et al. (2018) noted that most respondents (85.7%) preferred face-to-face services over electronic services for mental health, but almost 40% declared intentions to use the latter in the future. Many studies show that students readily express intentions to use and favorable attitudes toward online services (Farrer et al., 2013; Dunbar et al., 2018). Moreover, some authors point out that previous experience of (online) therapy helps to develop more positive attitudes toward online psychological help. Clients who participate in videoconferencing psychotherapy without previously meeting their psychologist in-person may question its’ credibility and effectiveness (Hall et al., 2022). Also, Dunbar et al. (2018) found that students with current severe psychological distress but no prior face-to-face treatment were less likely than those with a history of face-to-face treatment to endorse preferences for face-to-face services. Prior research suggests that certain attitudes can influence use of online psychological help among different groups of people, including patients. According to Apolinário-Hagen et al. (2018), some patients perceive online therapist interventions as helpful but not equal to face-to-face therapy. Patients who were more aware of the possibility of online therapy were more willing to use various forms of Internet-based psychological help. People experiencing stress preferred using guided online self-help over videoconference with a therapist. Those who developed an avoidant attachment style favored guided and unguided online self-help more than direct connection via the Internet with a therapist. McDonald et al. (2020) noted that some patients assessed online psychological help as an opportunity to be less inhibited, and more open to sharing information and establishing interactions. Cook and Doyle (2002) point out that people who feel insecure in social situations may view online contact as more secure and consequently feel more comfortable expressing their feelings more openly. Younger patients are more likely to use the Internet for therapy (McDonald et al., 2020), especially if they prefer to use the Internet in general for purposes other than therapy (Sweeney et al., 2019). Thus, the findings are not sufficiently detailed in terms of the experiences and attitudes. Results are also inconsistent, suggesting that different groups of patients (or clients) may see different benefits and risks of online psychological help. Moreover, attitudes toward, and beliefs about, online psychological help seem to be important for its’ effectiveness and trustworthiness. Schröder et al. (2015, 2018) point out that positive attitudes and beliefs are related to higher benefits and level of engagement in online psychological help. It was also observed that, in the case of suicidal thoughts, the effectiveness of online interventions is likely mainly related to program structure, monitoring, and safety procedures (van Spijker et al., 2018), which shows a commitment component, close to the acceptance of a given way of working on oneself. Considering the trustworthy issue, the literature suggests that lower online help credibility relates to psychologists’ lack of knowledge about the sociocultural context (e.g., Sampson et al., 1997), but it is not specific to online help (e.g., Sue and Sundberg, 1996; Zhang and Burkard, 2008). The Internet provides the opportunity to use the support provided by psychologists worldwide (e.g., speaking the same language but living in other countries). Consequently, it creates a greater risk that the psychologist will not be familiar with the cultural code the client/patient uses daily. Thus the psychologist may omit specific issues that could be important regarding the support provided. Some authors (Maples and Han, 2008) point out that the lack of face-to-face contact itself may be a reason for the lower credibility of the support offered online. However, some authors (e.g., Axelsson et al., 2020) indicate no differences in reliability between online and face-to-face assistance, which implies some inconsistency in this matter.

Our research considers the following lines of evidence (1) that there is an ongoing process of scientific recognition of the advantages and limitations of modern technologies applied in the field of psychological help, (2) attitudes are essential for the effectiveness of online help, so understanding these attitudes can support those responsible for providing online help, (3) the pandemic, as a vast contextual factor (e.g., social, economic, political), may have influenced attitudes toward online psychological help, so the question of the image of online psychological help that people present becomes particularly important, and (4) literature about online psychological help includes more research regarding the therapist/psychologist perspective, including their theoretical background, rather than patients or clients.

The aim of our research was to develop an understanding of experiencing online psychological help by potential beneficiaries, through analyzing participants’ voluntary statements from an online scientific psychological study. Our inquiry gives voice to the patients/clients and depicts their perception of online psychological help based on experiences, opinions, emotions, and attitudes. Online psychological help may be more than just a temporary solution for reduced access to face-to-face psychological help during the COVID-19 pandemic. Thus, the results of this study can help to (1) understand which patient/client attitudes are related to online psychological help and (2) improve the planning of online psychological interventions that could match its potential. We believe the presented findings would be helpful for a broad group of mental health workers and (potential) patients/clients.

## Method

The main objective of this study was to describe the perception of online psychological help in the experiences and opinions of people who were, or could become, beneficiaries of such help. Given the explorative goal of our study, we decided to use thematic analysis (Braun and Clarke, 2006, 2012) for analysis of written statements. We have assumed two approaches (1) a general phenomenological philosophical approach, concentrating on deep meanings and bracketing our experiences as researchers (Major and Savin-Baden, 2010) and (2) constructionist approach focused on meanings developed in peoples’ experiences (Crotty, 1998).

### Sampling

The statements about online psychological help were collected regarding an existing project about online psychological help. The following question was added at the end of the survey: *If you want to provide us with something about the relationships between the COVID-19 pandemic and online help/psychotherapy, please let us know below* The general aim of the primary study was to investigate the psychometric properties of the Attitudes Toward Psychological Online Interventions – Polish version (APOI-PL; Soroko et al., 2023 under revision in Frontiers; manuscript ID: 1168579). In the original study were recruited from a community sample (*N* = 304; 200 females, 99 males, 5 did not stated their gender; age *M* = 27,75; *SD* = 10,06). All data were collected at the beginning of the COVID-19 pandemic (March 2020 – September 2020). The online survey was disseminated via Facebook (paid advertisements) and participants were also recruited among researchers’ social networks via snowball selection. When asked what kind of online psychological help a person was referring to in the survey (question with many options to choose), we found that most people answered “online psychotherapy and counseling” (87.17%). Some people also indicated that for them, “online psychological help” is also web-based intervention (13.48%), internet-operated therapeutic software (13.81%), or other online activities (14.14%). Nearly a quarter of the total amount of participants (see Participants section) voluntarily decided to share their opinion. After reviewing the data, we noticed that online psychological help constitutes an unexplored and critical topic, which participants want to discuss or share their views of. We included all 72 written statements (words statistics: *M* = 57.83; *SD* = 51.76; *min* = 9; *max* = 320) in the analysis because they all contained at least one point that a participant wanted to convey. The statements were exclusively analyzed in the current study, as a separate data set.

### Participants

Participants (*N =* 72; 45 females, 25 males, 2 did not stated their gender; age *M =* 25; *SD =* 9.78; *min =* 19, *max =* 68) were individuals who voluntarily shared their experiences and attitudes about online help. Thirty-six participants declared current involvement in psychotherapy or other psychological help; twenty-eight declared current or past involvement in online psychological help; twenty-one stated that they benefit from pharmacotherapy to stabilize psychological functioning. Fifty-five participants elaborated on what type of online help they thought about when they shared their opinions and experiences. Forty-four of them talked about online help and psychotherapy, while the remainder indicated online apps or sites.

### Researchers

We are aware that qualitative research, including the results, could be affected by the authors’ perspectives. We therefore describe below all personal experiences or attitudes related to online contact during the COVID-19 pandemic, as well as additional information. The authors are Psychologists and Researchers associated with the *Qualitative and Mixed Method in Clinical Psychology Research Lab* at *Adam Mickiewicz University* in Poznań. At the time of this study, the first author (AW) was a PhD student and school psychologist with experience as being after-school club educator. During the COVID-19 pandemic, he took part in, and observed, online teaching in a primary school in Poland. In general, he perceived online education to be worse and less effective than in-person education. Moreover, he believed that the time that children spent in their houses may have had a harmful impact on their peer relationships, ability to cope with stress, negative emotions (e.g., anger or anxiety), and ability to focus their attention. Almost all children he talked to claimed that they prefer in-person education to online. These experiences and considerations about educational/online contact at school have increased his interest in attitudes toward online contact with mental health specialists. Thus, he monitored the differentiation of online psychological help and online education during the analysis of the present study. The second author (JS) was a psychology student, who graduated from the university during the analysis. Her own experiences considering online education made her interested in this research. Online classes at the university and during the Erasmus exchange negatively impacted her motivation, and she developed a tendency to isolate herself from others. Therefore, during the analysis, she had to be particularly careful so that her negative attitude toward living online did not affect the interpretation of the data. ES supervised this research. At the time of this study, ES also started to investigate psychodynamic psychotherapists’ adaptation toward online psychological help and was in the process of building her own attitude toward online psychotherapy. However, her starting point was the resistant perspective. Thus, she was very sensitive to the valence (positive vs. negative) of the content codes and themes. Considering the number of responses to the open-ended question on online help, which was a relatively new phenomenon in Poland, the authors were curious to know how online psychological help was perceived at the onset of the COVID-19 pandemic. All authors were motivated to review their views on online psychological help.

### Analysis

We applied the general phenomenological perspective to data analysis and used inductive thematic analysis according to the procedure formulated by Braun and Clarke (2006, 2012). This approach was dictated by the need to explore a relatively new phenomenon without any prior theoretic assumptions by evoking participants’ perspectives. Moreover, we believed that organizing qualitative data in themes (patterns) would allow us to reach the major meaning expressed by participants. Each statement was treated as a separate analytic unit. We analyzed the statements collaboratively (Richards and Hemphill, 2018), such that the first and second (named researchers in the next paragraph) authors consulted analytic decisions with the third author (supervisor) throughout the analytic process.

The steps of the procedure were as follows. First, each of the two researchers independently read all statements to familiarize themselves with the data, and then met to decide on a direction for analysis, specific aims, and research questions. The primary research question was: what experiences or attitudes about online psychological help do participants express? They coded both explicit extracts (descriptive coding) and implicit meaning (analytic coding; Gibbs, 2018). Second, each of the two researchers independently generated initial codes, and then met to share outcomes to check if their codes were accurate, understandable, and could be derived from the extract they were assigned to. The researchers and their supervisor then discussed and unified the code set and continued to work on an expanded code set. Third, each of the two researchers independently identified themes. Fourth, each of the two researchers independently prepared a list of candidates for themes that were revised in another collaborative meeting. Each researcher checked if every theme on their list created a coherent pattern, and if every theme was valid for the whole data set. Fifth, themes were defined according to each theme’s central meaning. A meeting was held to discuss and agree on a list of themes. The final wording of each theme was discussed and agreed upon during the meeting with the project supervisor. Sixth, the quotes were matched to themes and the most illustrative quotes were selected to report. During this phase, the themes were reorganized to develop a better narrative. To ensure data quality we employed strategies such as securing time to immerse in the dataset, researcher triangulation, independent coding with collaborative meetings, documentation of analytical decisions, and team consensus on themes (Nowell et al., 2017).

## Results

**Figure 1:**
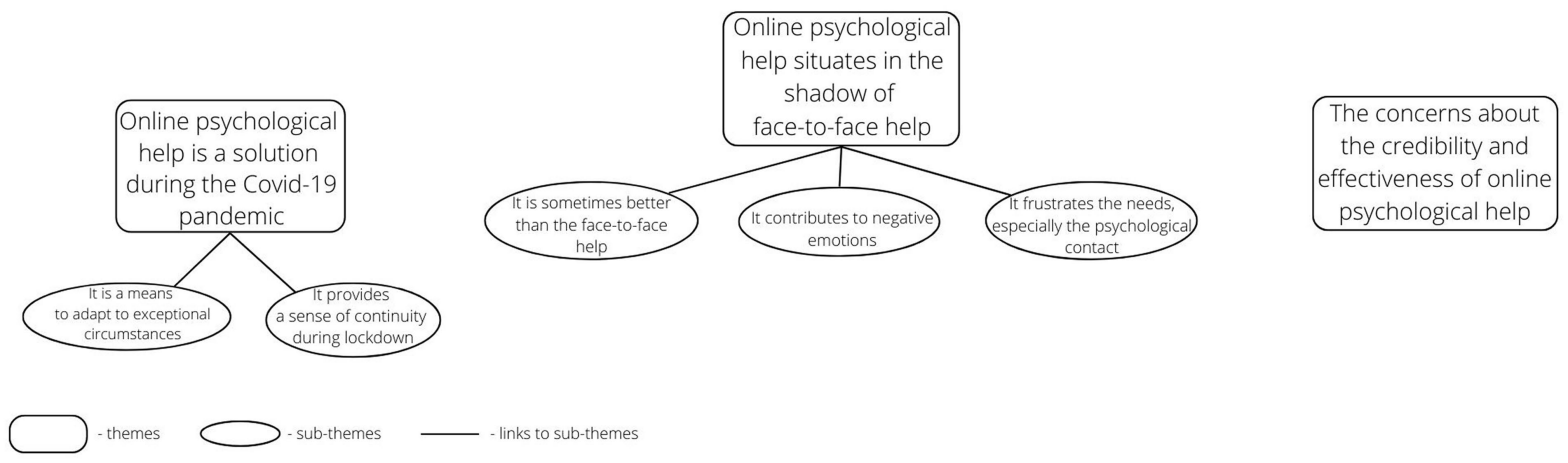
The thematic analysis map.

Three general themes with sub-themes were identified in the thematic analysis. The themes refer to meaningful separate aspects of online psychological help that are present in data in the scope of research questions (Figure 1; Table 1).

**Table 1:** The list of themes and sub-themes.

No	Themes and sub-themes	Percentage contribution of statements
1.	Online psychological help situates in the shadow of face-to-face help	
	1.1. It frustrates the needs, especially the need for psychological contact	30.6%
	1.2. It contributes to negative emotions	22.2%
	1.3. It is sometimes better than the face-to-face help	6.9%
2.	Online psychological help is a solution during the COVID-19 pandemic	
	2.1. It provides a sense of continuity during lockdown	27.7%
	2.2. It is a means to adapt to exceptional circumstances	30.6%
3.	The concerns about the credibility and effectiveness of online psychological help	25%

Research question: what experiences or attitudes about online psychological help do the participants express?

### Theme 1. Online psychological help situates in the shadow of face-to-face help

Participants generally made unfavorable comparisons between online and face-to-face psychological help and wrote about limitations of online psychological help relative to face-to-face help, especially considering the restraints of psychological contact and the therapeutic relationship. They indicated mostly negative aspects of online psychological help, which do not appear (in their opinion) during the face-to-face meetings. However, they also referred to the advantages of online psychological help.

#### Sub-theme 1.1. It frustrates the needs, especially the need for psychological contact

This theme refers to notions about a vast array of needs that cannot be satisfied by online meetings with a psychologist. According to participants, online psychological help frustrates, for an instant, a need for security, for physical/real contact, of communion and closeness, to be understood, or for a comfortable place to meet.

Online contact was perceived to be less natural than face-to-face contact, especially because of the lack of nonverbal communication that may jeopardize the objectives of psychological help. For example, one participant wrote: *It would be more difficult for the specialist to read the patient’s non-verbal signs* [while online meeting], and another: [online psychological help] *disrupts communication on a natural level.. well – it’s less natural (..), physical presence promotes therapy*. This participant alludes to the need for contact rooted in physical space and attributes it to a key role for effective help. Online contact with a psychologist was also referred to as dysfunctional, unnatural, and more challenging to engage with and maintain than face-to-face contact, as evidenced through the following two statements: *Online therapy is not like „live” meetings. Online work is worse, more difficult.* (…) *you cannot keep eye contact with therapist, it’s difficult to work some topics over without face-to-face contact* and *Whether in real life or in online therapy, confidentiality and a therapeutic relationship are important. In online therapy, confidentiality is assured, but the therapeutic relationship is more difficult to make.*

There were also some statements about the perceived challenge of opening up during online help, e.g., *I sometimes feel that I would rather see a psychotherapist in person and that it would be easier to talk about some of my problems*. Participants also spoke on behalf of other people, such as those suffering from schizophrenia, as they perceived certain groups would never able to adapt to online help and they would *inevitably require face-to-face care and contact with a specialist*.

Moreover, participants revealed the need of the psychotherapist’s office as a place where one can feel secure. A need for security was expressed, as well as concerns about privacy deprivation and lack of confidentiality as in the following statement: *The conversation is accompanied by fears that a member of the household might overhear something very intimate*, and another: (…) *if you do not live alone, you may feel uncomfortable and be dishonest because of fear of being overheard. Home is not always a safe place*.

#### Sub-theme 1.2. It contributes to negative emotions

Participants mentioned negative emotions that they had experienced with online psychological help, predominantly fear, anxiety, and anger (see also Sub-theme 1.3 for positive emotions). Negative emotions were usually related to the frustration of needs, mentioned above, due in part to the uncontrolled conditions of online meetings but that are absent in face-to-face appointments (e.g., behavior of others in the household or technical difficulties). The following quotes illustrate fear and anxiety related to others in the household: *When using online help, it can be difficult to open up if you live with other people because there is a fear of being heard*, and another: *The conversation is accompanied by fears about whether any member of the household will accidentally hear something very intimate* (…).

However, not all negative emotions seem to be induced by frustrated needs. Some participants stated that online appointments are more stressful than face-to-face, e.g., *The transition from traditional, or face-to-face, therapy to online therapy is quite a stressful event for many people. Many people I know feel tremendous stress, even more than during a regular visit*. Their negative emotions were related to uncontrolled technical issues: *I also feel the fear of technical problems that may affect the quality of the online meeting (…)*; or general dissatisfaction related to the transition from face-to-face to online therapy, e.g., *Because of my psychological well-being, I decided to continue therapy online. I am not happy about it (..)*

Some also pointed out uncertainty about whether help can be provided professionally online. One participant said: *Professionals do not necessarily know how to help others through a phone or computer screen*. Another participant expressed anger, saying, [online psychological help] *is terrible, during online consultation I feel ignored, belittled‚ mute*!

#### Sub-theme 1.3. It is sometimes better than face-to-face help

In the participants’ statements, we also observed some comparisons made in favor of online help. Participants claimed that online help could resolve some economic or transportation problems, such as driving to the psychological clinic for those who live in small towns or villages, e.g., *to people having difficulty traveling to a larger city*. One participant described how online help could be an antidote to overcome limited access to healthcare: *Many people do not have access to a psychotherapist or psychiatrist close to where they live, and such online help is de facto the only help they can afford if they cannot see a specialist in person. A*nother participant highlighted the opportunity to support people with various disabilities, e.g., *better solution for those with various disabilities*.

Moreover, online psychological help seems to reduce fear and anxiety related to revealing the fact of being in therapy to others. The online nature of the meetings helps to maintain confidentiality, e.g., *It is good that it is possible to meet a psychologist/psychotherapist via the Internet because patients do not have to worry that someone will see them when they enter the psychologist’s clinic*. Participants also mentioned relief, happiness, or pleasure in relation to the possibility to continue having contact with a psychologist/psychotherapist during the pandemic, e.g., *For me* [online psychological help] *works well, I’m happy with the results, and I also think it’s better to continue online therapy and try to convince myself rather than abandon it completely.*

### Theme 2. Online psychological help is a solution during the COVID-19 pandemic

Participants perceived that online psychological help was a forced adaptation to the COVID-19 pandemic that should be reversed as soon as possible so as to avoid negatively impacting psychotherapy as a professional activity. Here, we identified two sub-themes: providing a sense of continuity during lockdown and adapting to exceptional circumstances.

#### Sub-theme 2.1. It provides a sense of continuity during lockdown

According to research participants, online psychological help provides a sense of continuity in the use of psychological help that had been previously started in a face-to-face setting. Participants, who referred to their own psychotherapy process, experienced a forced choice between online meetings or to cease/suspend current contact. The involuntary aspect was highlighted, e.g., *No possibility of “live” meetings!*; *No therapy is worse than online therapy when there is no other option*.

Some participants described online psychological help as the only safe option to maintain contact with a psychologist or psychotherapist in the pandemic and lockdown. Some participants referred to the experience of moving from face-to-face to online contact, e.g., *My psychotherapy began in the office but was interrupted by a lockdown. Very quickly, the therapist suggested online meetings, initially as a “maintenance of the therapeutic relationship.”* Other participants reported that online help during the pandemic was widely needed, and there were people who experienced abandonment by therapists, particularly when they did not switch to online help, and were left alone with intense emotions, e.g., (…) *when faced with canceled appointments – they do not know what to do, where to go, who will help them. Patients left on their own cannot cope because their original problem/disorder is joined by panic attacks, hysterical crying.* The potential lack of any psychological help (during the pandemic) was perceived to be hard to cope with on a daily basis, e.g., *I am frightened by the vision of not having therapy and the help of a psychiatrist in my everyday life.*

#### Sub-theme 2.2. It is a means to adapt to exceptional circumstances

At the time of data collection, for many participants, online psychological help was a temporary adaptation, e.g., *I had to switch to this form of therapy in these strange times. But ultimately I’m supposed to have psychotherapy in real life*. For others, online psychological help was a more permanent means of communication between patient and therapist, which could regularly act as *a substitute in crisis situations,* at least for certain groups of people in need, e.g., *only an emergency form of assistance for the bereaved*. Participants elaborated on the specific conditions under which online psychological help is more desirable, e.g., *if online psychotherapy is conducted by a professional, and the choice of this form is not due to a desire to avoid involvement in one’s psychotherapeutic process, but is due to objective reasons* (e.g.*, quarantine, lack of a Polish-speaking psychotherapist in the area*)*, then it makes sense and is needed*.

Online psychological help was also recognized as an effect of pecuniary or organizational adjustment, ultimately mitigating the impact of COVID-19 on people and the economy. One participant wrote: *If it were not for the fact that it was possible to enforce remote work during the epidemic situation using ICT tools, it would have been a disaster. Financially -for the National Health Service, therapists and clinic staff, and most of all for patients, who would lose the possibility of support and continuity of therapy*. Another participant using English (potentially to express cosmopolitan feelings) demonstrated gratitude or relief: *Thank gods for the internet!* In the longer term, experiences with online psychological help that have been accelerated by the COVID-19 pandemic may affect professional help in a general sense, especially by *gaining a wider reach than if there were the absence of a pandemic threat.*

### Theme 3. The concerns about the credibility and effectiveness of online psychological help

Some participants expressed concerns about the effectiveness of online psychological help or its’ credibility. The statements draw attention to the difficulty in verifying the credibility of the psychologist, the risks of seeking help from an unreliable source (e.g., an online forum or a website of unknown origin), and sometimes to direct harmfulness from non-professional groups, which are difficult for lay people to distinguish from professional groups due to advertising or positioning. Many participants also expressed their concerns or doubts about the effectiveness of online psychological help.

A lack of confidence toward psychotherapists working online was also expressed. The lack of control and the impossibility of discovering the truth about professionalism of the therapist, and the interplay between easy access and restricted service quality, were indicated, e.g., *The downside is that anyone can advertise as a therapist, and it is easy to come across a charlatan.* The image of a self-regarding psychologist was also described, e.g., *offering psychological help to people in crisis is more therapeutic for the psychologist him/herself, who also feels anxiety and tries to deal with it by being useful*.

Attention was also drawn to online groups, which can be confused with receiving online professional help. Online groups may look similar to a place where people can get psychological support from people commenting on online posts. However, *the comment is made by an ordinary Kowalski* [Mr. Smith] *who has no psychological training and his advice does not help the person concerned, it can only harm him.* Even if there are psychologists in support groups, they are *random people from the Internet who do not know you intimately.* In addition, *in front of the computer it is easier to pretend,* so *it is easy to sweep some* (*key*) *problems under the carpet*. Online psychological help can offer the illusion of help by misleading those most in need, e.g., *therapeutic apps of all kinds are usually bullshit, possibly helping people who, despite their problems, have no problem with motivation and regularity*).

Moreover, without professional care, there can be some social viral processes that can impede coping with the pandemic and other crisis. Indicating an *increasing number of posts tinged with panic, anxiety, and questions about how others deal with panic. As this is not a group of psychologists, these people do not get reliable answers, but ones that “feed” the panic.* The lack of a Psychologist can increase the harmfulness of online groups. Thus, the potential harm of online groups or forums mainly concerns mistaking them for professional help, and some are not sufficient for reaching health benefits. However, in some cases, *forums like Quora or Facebook groups can serve as a good source of psychological knowledge, but for individual work, regular therapy is definitely more helpful*.

Many participants also expressed their concerns about the effectiveness of online psychological help. Some have questioned the efficacy of online psychological help in therapy for specific disorders, e.g., (…) *With schizophrenia and other similar disorders, I think such* [online] *help would not be very successful*. Others are (…) *not sure that online meetings with a person you meet online will be as effective* [as face-to-face help]. Consequently, the participants tend to undermine the effectiveness of online psychological help in general, e.g., (…) *people who have already been diagnosed with mental problems, the symptoms will get worse or remain at the same level regardless of online help*, or *Psychotherapy over the Internet due to the epidemic situation 90 percent of the time does not meet the help that a psychologist*, *psychotherapist would offer in the office*.

## Discussion

Our study aimed to develop an understanding of experiencing online psychological help by (potential) patients and clients form a community sample. It is important to note that the study occurred at the beginning of the COVID-19 pandemic in Poland. Using thematic analysis, which we embedded in a phenomenological and constructionist approach, we analyzed the voluntary statements of 72 research participants. Three main themes, along with sub-themes, were identified, addressing the question of what attitudes and experiences of online psychological help are expressed by the participants (see Table 1 and Figure 1). Below we discuss the themes and present the practical implications of the findings, in order to improve the planning of future online psychological interventions.

Our study found that online psychological help situates in the shadow of face-to-face psychological help. We identified an apparent viewpoint that online psychological help frustrates the needs of patients, especially the need for psychological contact, and that online psychological help contributes to negative emotions but is sometimes better than face-to-face help. Online help is discussed in the context of face-to-face help. People treat online psychological help, adequate to its preceding place in health care, as secondary and derivative to face-to-face psychological help. Our findings show that comparing online and face-to-face help is common and often is not in favor of online therapy, especially regarding the quality of the help and the frustration of the range of relational needs, resulting in negative feelings, mainly anxiety, anger, and disappointment. This image of online psychological help can discourage people from engaging in it, even when it would be advisable. As mentioned previously (Farrer et al., 2013; Dunbar et al., 2018; March et al., 2018; Hall et al., 2022), research on attitudes toward online interventions are inconsistent; however, in our community sample of young participants, we identified the presence of a rather negative attitude. This may be related to the timing of the study (the onset of the pandemic) in tandem with the need to adapt ones’ views to changes in health care (the transition of private practices and national centers to remote interventions), which may have radicalized and hardened views. Moreover, limited experience in using face-to-face help before a first online psychological appointment, which was also present in our sample, may favor the idealization of face-to-face contact and reduce openness.

Online contact proves particularly desirable, but only under certain conditions. Advantages are mainly considered in the context of overcoming the limited access to healthcare resulting from objective factors (e.g., geographic, economic, disability), which is often discussed in the literature (Clarke et al., 2009; Stoll et al., 2019). Better conditions for anonymity and privacy are also indicated, and perceived, or actual, anonymity may lead to reduced inhibition and, in turn, greater openness in discussing emotional topics (see more in Stoll et al., 2019). On addition, from a technological point of view (e.g., unsecured websites or unencrypted communication tools), the privacy, confidentiality, and security issue of e-health are overrated (Stoll et al., 2019). The issue of anonymity may be particularly important for shaping attitudes: on the one hand, patients may truly benefit from greater anonymity, but on the other hand, it is informative of the fear of stigma present in society as a result of using health care and may require anti-stigma interventions, such as education combining social contact (Lien et al., 2021), but also technology education.

One such objective factor relevant to attitudes toward online psychological help was the COVID-19 pandemic. One could say that we captured a moment of attitude change in terms of recognizing the suitability of online psychological help. Participants demonstrated that online psychological help is one type of solution because it provides a sense of continuity during lockdown and is a means to adapt to exceptional circumstances. Thanks to online help, the continuity of the therapeutic relationship could be maintained after the disruptive transition to remote therapies. In turn, internet-based interventions, in a situation of a pandemic-related mental health crisis, were able to begin to support self-care in the area of mental health for many patients. While the potential of online psychological interventions has been recognized, its uncertainties and limitations have been pointed out (e.g., only maintenance psychotherapy, lack of accessibility for those suffering from severe mental disorders, or when therapists refuse to switch to an online form and stop contact). This shows the exclusivity still present in the e-mental health field, in that availability and access to online psychological help is both an advantage and disadvantage, but directed toward different groups of potential beneficiaries (Stoll et al., 2019).

Participants recognized online psychological help as a means to adapt to exceptional circumstances, paying attention to its provisionality. Issues of temporality were also observed in research about the transition from face-to-face to online treatment for eating disorders (Lewis et al., 2021). Most participants (68%) stated that they would not choose to continue online therapy given the option after lockdown. Factors such as higher COVID-19 anxiety, longer duration of treatment, and stronger therapeutic alliance were associated with more positive views toward the permanent transition to the online form.

Our findings indicated that the help provided online was the result of a process of adaptation, both among patients (who expressed gratitude for the possibility of contact or the availability of options beyond face-to-face contact) as well as therapists and the overall environment in which mental health care organization occurs. In the recent literature, we find descriptions of these complex processes considering psychotherapists and centers (Sasangohar et al., 2020; Vostanis and Bell, 2020; Green et al., 2021), but we did not find literature on the process of patients’ adaptation to online help. Meanwhile, the need for self-determination and to develop one’s own opinions is evident in the statements of the participants in our study. We have identified the expression of conscious acceptance of a temporary form of therapeutic contact or sensitivity to patients who would not benefit from such a form. The apparent sensitivity to others may manifest altruistic attitudes noted in pandemic-threatening situations that have been shown to influence prosocial and altruistic behavior (Grimalda et al., 2021) and increased loyalty in the social groups of patients, which may be a socially new phenomenon.

In our research, participants referred to the fact that online psychological help could become harmful in certain circumstances. Participants drew attention to the and uncertainty of who was on the other side of the computer (mainly processes that were initiated via the Internet), and also to the risk of receiving unprofessional advice on the Internet, for example, in groups or forums, or even treating advice erroneously as psychological help. In research on help and psychotherapy, the study of adverse or detrimental effects is rarely addressed (Klatte et al., 2022). In our case, we encountered an occupation with a concern about the detrimental effects of help, stemming from knowledge about the functioning of the Internet, especially social media, which can pose a threat to the public and individual health, e.g., by facilitating the spread of misinformation or anonymous, hateful comments (Walter et al., 2021). Critical thinking and caution toward supportive content on the Internet were present. There is an apparent need for the community to critically consider and develop strategies to counteract the true-to-life limitations of online psychological help. For professionals, this signals the need to develop and promulgate standards for professional online help.

### Practical implications

The experiences, attitudes, and opinions about online psychological help, as captured in the themes and sub-themes presented above, allow us to make some practical implications for mental health professionals. Internet interventions might help to bridge the large treatment gap, but should be well-introduced with regard to evidence-based knowledge. The availability of face-to-face psychological support is still limited worldwide. For example, Schröder et al. (2018) showed that only a minority of people suffering from depression receive adequate treatment. Scientific evidence is emerging that online psychological help is a good solution to extend the reach of mental health care by introducing internet-based interventions, especially for patients with depression or substance misuse (Fu et al., 2020). Therefore, attempts are being made to better understand factors that might impede or facilitate the use of these services, and questions are open on how to best encourage translation of intentions to use online psychological help into behavior (March et al., 2018). Some data show that enhancing confidence and familiarity with technology might be the first step. Others suggest that, to meet the needs of youth, in-person options and diverse, accessible, technologically stable virtual services are required (Hawke et al., 2021). Solutions such as a stepped-care approach to treatment are also being introduced, as an example of increasing the efficiency of available mental healthcare resources (Ong et al., 2021). Our study also suggests that it is important to support clients’, patients’, or beneficiaries’ self-understanding (e.g., emotions), especially in the context of expectations of the therapeutic relationship in online psychological help. With well-managed and transparent rules, disappointment and other negative emotions could be reduced, and reaching out for online help may be more reasonable, e.g., matching the type of intervention to the patient’s problem or using online psychological help only in certain circumstances that are acceptable to the patient. Our research suggests that online mental health care in social, climate, and energy crises may also be gaining recognition, as the process of becoming accustomed to online solutions has begun and online solutions are being acknowledged as reasonable.

Psychological services meant to be conducted online require clear, and perhaps distinct but not necessarily more liberal, criteria for assessing their quality. People need more sound knowledge in order to develop a better idea of the quality of the online help offered and its potential range of helpfulness, or usefulness, without attributing it as a panacea for every problematic condition and without instinctively rejecting it. Patients highly value a low price and personal contact with a psychotherapist, as well as proven effectiveness (Phillips et al., 2021); thus, there is a requirement to disseminate the results of research into the effectiveness of individual online assistance programs and to familiarize people with technological aspects. Standardized (Fu et al., 2020) but also client-informed implementation of online psychological interventions are necessary.

A notable reflection on the professionalization of online support has been undertaken on using mobile apps for mental health (Marques et al., 2021). It was shown not only that mobile apps are not suitable for all psychological issues, but also that mental healthcare professionals should be involved in co-designing these apps and apply suitable psychological theories (e.g., cognitive-behavioral). Another suggestion is to develop and share guidelines to evaluate mental care mobile apps and incorporate the citation of sources and privacy information to the end-users. Similar processes of quality care and psycho-education are needed on a large scale.

### Limitations and future directions

According to the criteria of quality qualitative research (Flick, 2018), our findings cannot be generalized but rather understood from the perspective of the respondents’ specific social and psychological situations. The study participants took part in the questionnaire survey but additionally wished to share their experiences and opinions. A significant limitation is that the circumstances of their motivation remain unknown to us. Furthermore, we can only guess from the emotionality of the statements that it was an opportunity for participants to express their forming views or regulate their emotions. In turn, we infer from the temporal timing of the survey that these attitudes are about online assistance expressed at a moment of intense transformation, so people were sharing hotly-formed views on an ongoing basis. We are not in a position to determine the validity of the image of online help we received, but only to describe those thematic areas that we recognize as significant for the “destiny” of online psychological help now and in the future.

In addition, the opinions and experiences of our participants ranged widely in terms of online psychological help, including online psychotherapy (which had previously been conducted face-to-face, and one which included remote contact from the beginning of the process), online counseling, mental care mobile apps, or step-by-step programs. Further research should undoubtedly focus on attitudes toward specific forms of online help, especially self-help, so that we can begin to differentiate attitudes better.

Moreover, the time of data collection could be perceived as one of the study’s limitations. On the one hand, data collected at the beginning of the COVID-19 allows interpreting the results exclusively as a picture of attitudes toward online psychological help precisely during the intensive shift (different both from the pre-pandemic period and today) face-to-face to online contact. On the other hand, the presented research could induce further studies on current attitudes in order to make an attempt to show how people have been adapting to online psychological help during more than 2 years of the pandemic. Thus, the outcomes could capture changes in attitudes toward this kind of psychological help.

Another limitation is the fact that the sample was not recruited specifically for the current study. The participants are nearly a quarter of the total number of participants who took part in different research investigating psychometric properties of the APOI-PL and voluntarily shared their opinions about online psychological help in open-ended question at the end of the survey. Therefore, the motives for answering the questions are not known. Given the studys’ overall conclusion (negative rather than positive opinions, emotions, and attitudes), it is possible that those who answered the question primarily wanted to share their concerns. Accordingly, it would be fruitful to design a study to investigate precisely attitudes toward online psychological help. Thus the results could present the attitudes in a broader and more comprehensive perspective.

Not all participants declared current or past involvement in therapy or other forms of online psychological help. Consequently, our results include attitudes developed both from their own experience and opinions and information that participants heard from others or read on the Internet. Further study could focus on attitudes toward those with and without experience of participating in online psychological help, attempt to determine their origins, and compare them (e.g., which are more positive/negative and why).

Philosophical assumptions allowed us to adopt a non-evaluative position toward the participants, in that we successfully bracketed our preconceptions and stayed by the participant. At the same time, the potential of phenomenology as an approach could not be fully exploited because we analyzed foundational data. Further research, therefore, is needed to explore attitudes toward online therapy using in-depth interviews, whereby researchers can explore how participants’ experiences are organized before they are fully categorized.

The analytical technique (thematic analysis) has apparent limitations, but it allowed us to recognize patterns in the data. In our case, these were the major themes of attitudes toward online psychological help, which we extracted through our engagement with the topic as a research team. We, therefore, take full responsibility for the story we heard from the subjects. We presented in the report what we read from participants who took the opportunity to share statements about online psychological help or discuss the phenomenon. This article gave them a voice and let both (potential) beneficiaries and caregivers know more about attitudes, concerns, and general experiences related to online psychological help in the context of pandemic experiences.

## Conclusion

In summary, if the effectiveness of online psychological help is influenced by attitudes toward it, the current findings support practice, especially the practice of mental health care professionals. The results show the attitudes, emotions, thoughts, hopes, concerns, and limitations that are related to current or imagined involvement in online psychological help as a patient (client). Awareness of these perspectives could help to improve the planning of online psychological interventions, taking into account potential difficulties that patients face. Moreover, our results could allow (potential) patients/clients who are considering the use of online psychological help to view other people’s feelings about such services.

## Data availability statement

The raw data supporting the conclusions of this article will be made available by the authors, without undue reservation.

## Ethics statement

Ethical review and approval was not required for the study on human participants in accordance with the local legislation and institutional requirements. The patients/participants provided their written informed consent to participate in this study.

## Author contributions

AW, JS, and ES were responsible for research conceptualization. AW and JS contributed to the datasets’ formal analysis. AW was the project administrator and managed the thematic analysis process, which ES supervised. AW and JS wrote the original draft. ES and AW reviewed and edited the original version. AW visualized the thematic analysis map. All authors contributed to the article and approved the submitted version.

## Conflict of interest

The authors declare that the research was conducted in the absence of any commercial or financial relationships that could be construed as a potential conflict of interest.

## Publisher’s note

All claims expressed in this article are solely those of the authors and do not necessarily represent those of their affiliated organizations, or those of the publisher, the editors and the reviewers. Any product that may be evaluated in this article, or claim that may be made by its manufacturer, is not guaranteed or endorsed by the publisher.
